# Identification of novel neuroprotectants against vincristine-induced neurotoxicity in iPSC-derived neurons

**DOI:** 10.1007/s00018-024-05340-x

**Published:** 2024-07-27

**Authors:** Veselina Petrova, Andrew R. Snavely, Jennifer Splaine, Shannon Zhen, Bhagat Singh, Roshan Pandey, Kuchuan Chen, Anya Cheng, Crystal Hermawan, Lee B. Barrett, Jennifer A. Smith, Clifford J. Woolf

**Affiliations:** 1 F.M. Kirby Neurobiology Center, Program in Neurobiology, Boston Children’s Hospital, Boston, MA 02115 USA; 2 Department of Neurobiology, Harvard Medical School, Boston, MA 02115 USA; 3 ICCB-Longwood Screening Facility, Harvard Medical School, 250 Longwood Avenue, Boston, MA 02115 USA; 4 F.M. Kirby Neurobiology Center, Center for Life Science, 3 Blackfan Circle, Boston, MA 02115 USA

**Keywords:** Chemotherapy, Vincristine-induced growth deficits, High-throughput screening, Neuroprotective small molecules, Human motor and sensory neurons

## Abstract

Chemotherapy-induced peripheral neuropathy (CIPN) is a disabling side effect of cancer chemotherapy that can often limit treatment options for cancer patients or have life-long neurodegenerative consequences that reduce the patient’s quality of life. CIPN is caused by the detrimental actions of various chemotherapeutic agents on peripheral axons. Currently, there are no approved preventative measures or treatment options for CIPN, highlighting the need for the discovery of novel therapeutics and improving our understanding of disease mechanisms. In this study, we utilized human-induced pluripotent stem cell (hiPSC)-derived motor neurons as a platform to mimic axonal damage after treatment with vincristine, a chemotherapeutic used for the treatment of breast cancers, osteosarcomas, and leukemia. We screened a total of 1902 small molecules for neuroprotective properties in rescuing vincristine-induced axon growth deficits. From our primary screen, we identified 38 hit compounds that were subjected to secondary dose response screens. Six compounds showed favorable pharmacological profiles – AZD7762, A-674563, Blebbistatin, Glesatinib, KW-2449, and Pelitinib, all novel neuroprotectants against vincristine toxicity to neurons. In addition, four of these six compounds also showed efficacy against vincristine-induced growth arrest in human iPSC-derived sensory neurons. In this study, we utilized high-throughput screening of a large library of compounds in a therapeutically relevant assay. We identified several novel compounds that are efficacious in protecting different neuronal subtypes from the toxicity induced by a common chemotherapeutic agent, vincristine which could have therapeutic potential in the clinic.

**Supplementary Information:**

The online version contains supplementary material available at 10.1007/s00018-024-05340-x.

## Introduction

Chemotherapeutic agents often have deleterious side effects that greatly reduce the quality of life of cancer patients. In many cases, these adverse effects lead to dose reduction or treatment termination, which worsens clinical outcomes. Chemotherapy-induced peripheral neuropathy (CIPN) results from damage to sensory and motor neurons by anti-cancer agents [1]. Symptoms include heightened sensitivity to stimuli, numbness, and tingling in the extremities, and, in some patients, chronic neuropathic pain [2]. CIPN occurs in 30–70% of patients [3], yet no established preventative or curative methods exist.

Studies have identified six groups of anti-cancer agents that cause peripheral neurodegeneration and consequent CIPN, including vinca alkaloids. Vinca alkaloids, a family of microtubule targeting agents, are widely used for the treatment of a variety of cancers, such as breast cancer, osteosarcomas, and leukemias [4]. As a primary mechanism of action, these drugs are anti-mitotic. Specifically, they inhibit the polymerization of microtubules by binding to tubulin proteins and preventing their phosphorylation [4], which arrests cancer cell proliferation before the onset of anaphase and prevents cell division.

Vincristine is among the most effective and widely used of the vinca alkaloids. It has been approved to treat various leukemias and lymphomas. However, it has also been found to be effective in off-label uses for treating many other cancers. Despite its efficacy, like most chemotherapeutics, clinical use of vincristine is often limited by its severe neurotoxicity in a subset of patients. In the clinic, vincristine-induced peripheral neuropathy has been observed to be dependent on vincristine’s cumulative dose and treatment intensity. For example, the typical single dose of vincristine is 1.4-1.6mg/m^2^ [5], yet multiple doses are often required, and significant symptoms of peripheral neuropathy can appear with a cumulative dose of 2–12 mg/m^2^ [6, 7]. Vincristine treatment is most associated with distal sensory and motor symptoms and, in some cases, autonomic disturbances.

Sensory symptoms such as tingling, numbness, loss of touch sensation, and neuropathic pain occur in a distal to proximal manner from days to weeks after vincristine treatment onset [8]. These symptoms are the first to appear and are those most commonly associated with vincristine treatment. In a small number of patients, motor symptoms such as muscle weakness in the extremities, wrist, and foot drop, and posture and balance deficiencies also occur [8, 9]. All these symptoms could significantly reduce the quality of life of cancer patients which stresses the importance of finding reliable models to identify neuroprotectants in both sensory and motor neuron subpopulations. In addition, vinca alkaloids have previously been shown to induce neuronal toxicity in both sensory and motor neurons through common mechanisms such as microtubule destabilization, axon transport deficits, mitochondrial defects, and oxidative stress [3], suggesting that newly identified neuroprotective compounds might be protective across multiple cell types.

There are several models using iPSC-derived sensory neurons that mimic specific CIPN characteristics. When testing multiple chemotherapeutic agents in one of these models, vincristine consistently has the most neurotoxic effect when compared to compounds like paclitaxel and bortezomib [10]. The neurotoxic vincristine phenotype includes axonal blebbing, axonal fragmentation [11] and arrested axonal outgrowth [12]. Studies have used this model to test chemotherapeutic drugs in a high-throughput assay format [13]. Additionally, this model has been used to identify signaling pathways associated with vincristine neurotoxicity, such as the phosphorylation of JNK [14]. However, there has not yet been a high-throughput screen with human iPSC-derived neurons that have reliably identified neuroprotective molecules.

In this study, we developed an assay using human iPSC-derived motor neurons suitable for high-throughput screening based on axon growth arrest 24 h post-treatment with 3 nM vincristine. We then performed a high-throughput screen with 1902 bioactive small molecules and identified six compounds that showed dose-dependent neuroprotection in this assay. Four compounds were also neuroprotective against vincristine in human iPSC-derived sensory neurons, indicating that the screen identifies neuroprotective compounds that act on multiple neuronal cell types and could, therefore, have therapeutic relevance.

## Materials and methods

### iPSC culture

iPSCs were maintained on a Geltrex LDEV-free matrix (ThemoFischer) in StemFlex (ThermoFischer). Cells were passaged at a ratio of 1:12 every four to six days, at approximately 80% confluence, using ReLeSR (Stem Cell Technologies) enzyme-free passaging reagent. After 10 passages, a new vial of iPSCs was thawed, or the established culture karyotyped. Human iPSC line SAH-0047-02 (from a 46-year-old, female donor) was graciously provided by the Sahin Laboratory, Boston Children’s Hospital, Boston, MA. Human iPSC line LiPSC-GR1.1 (from a male white Hispanic donor, age: <1D) was purchased from Lonza Inc. and used for sensory neuron differentiation.

### Motor neuron differentiation

iPSCs were differentiated into motor neurons as previously described [15]. Briefly, iPSCs were split into single cells using Accutase (Stem Cell Technologies) and plated at a density of 5 × 10^5^ cells per mL in a 10 cm Geltrex coated plate in StemFlex media with 1µM Y-27632. After the first day, cells were maintained in motor neuron media, a 50/50 mixture of DMEM F12 (ThermoFischer) and Neurobasal media (ThermoFischer) supplemented with B27 (ThermoFischer), N2 (ThermoFischer), Glutamax (ThermoFischer) and non-essential amino acids (Corning) as per the manufacturer’s recommendations. For the first five days, motor neuron media was supplemented with SB431542 (10µM) and LDN (100nM) to neutralize the cells and Retinoic acid (1µM) and SAG (1µM) to posteriorize and ventralize the cells. SB43152 and LDN were replaced on the sixth day with SU5402 (4µM) and DAPT (5µM) to specify motor neuron identity. After an additional 10 days, the cells were harvested by incubation with Accutase at 37^o^C for 45 min. The detached cells were washed twice with DMSO and filtered through a 70µM cell strainer before proceeding with motor neuron sorting or freezing in a 1:1 mixture of motor neuron media and Cyrostor CS10 (Stem Cell Technologies) in liquid nitrogen for future use.

Before each assay, motor neurons were thawed (when used from frozen) and sorted for CD56 (NCAM-1) expression. The thawed cells were resuspended at 1 × 10^8^ per mL in MACS buffer (0.5% BSA, 1mM EDTA in PBS) with 1/10th volume PE-conjugated anti-CD56 antibody (BD Bioscience #555516). Cells were incubated with the antibody on ice for 30 min before washing once with MACS buffer and resuspending in MACS buffer with 1/10th volume Anti-R-Phycoerythrin Magnetic Particles (BD Bioscience #557899). Cells and secondary antibodies were incubated at room temperature for 45 min. Bead-cell conjugates were captured by flowing the cell suspension through large cell MACS columns (Miltenyi Biotech #130-042-202). After elution from the columns, cells were counted and plated in motor neuron media supplemented with growth factors (35 µg/mL Ascorbic acid, 10ng/mL recombinant human BDNF, 10ng/mL recombinant human GDNF, 10ng/mL recombinant human CNTF) on plates coated with poly-D-lysine and laminin at a density appropriate for the assay.

### Sensory neuron differentiation

Sensory neuron differentiation was performed according to previously published protocols [16]. Briefly, iPSC cells were grown in Stemflex media and passaged for a total of 3–4 passages before onset of differentiation. IPSC cells were dissociated using Accutase and plated at 1.5 M cells/well density on 6-well plates in Stemflex with CEPT [17]. When 90% confluent, cells were treated with differentiation media containing E6 media, 0.2 µM CHIR-98,014 (SelleckChem, cat no. S2745) and 2 µM A83-01 (Tocris, cat no. 2939/10) for 3 days. Then, cells were replated at 5.5 M/cell in 6-well AgreWell Plates to stimulate the formation of nocispheres. Cells were treated with differentiation media containing E6 media, 0.5 µM CHIR-98014, 2 µM A83-01, 1 µM DBZ (Tocris, cat no. 4489/10) and 25 nM PD173074 (Tocris, cat no. 3044/10) for further 11 days. Nocispheres were then collected and dissociated using the MACS Embrioid Body Dissociation kit into a single cell suspension, plated on 384-well plates, and grown in neuronal media (DMEM/F12, B27 supplement, N2 supplement, 25 ng/mL BDNF, 25 ng/mL GDNF, 25 ng/mL β-NGF, 25 ng/mL NT-3, 1 µM PD-0332991 (Tocris, cat no. 4786/10).

### Small molecule screening

Induced motor neurons were plated at a density of 2,000 cells per well in poly-D-lysine coated 384-well plates 20 h before the beginning of the screening process. After 20 h, test compounds were added to the cells by diluting 100 nL of 3.3 mM drug into 30 uL media for a final assay concentration of 10µM. After 4 h of incubation at 37^o^C, 1/10th volume of 30nM vincristine in neuronal culture media was added to each well (except for 16 control wells per plate, which received an equivalent volume DMSO). The final concentration of vincristine in each well was 3 nM. Cells were then incubated at 37^o^C for 24 h. Cells were then fixed in 4% PFA for 25 min and stained for β-III-tubulin. Imaging and neurite quantification were performed using the neuronal profiling module of the Thermo Fisher Arrayscan XTi. All primary and secondary screens in motor neurons were carried out in duplicate.

Induced sensory neurons were plated at a density of 5000 cells per well in poly-D-lysine coated 384-well plates 20 h before screening. After 20 h, the top six rescue compounds identified from the motor neurons screen were added. After 4 h of incubation, 1 nM vincristine was added. Cells were then incubated at 37^o^C for 24 h. Cells were then fixed in 4% PFA for 25 min and stained for β-III-tubulin. Imaging was performed using Molecular Devices ImageXpress Micro Confocal High-Content Imaging System, and neurite quantification was performed using automated neurite tracking on the MetaExpress software. All sensory neuron screening was carried out in triplicate.

### Compound library

Screening was performed using the Selleck Bioactive small molecule library at the ICCB-Longwood Screening Facility at Harvard Medical School, Boston, MA. The library consisted of 1902 small molecules selected based on their known biological activity. 10mM stocks of compounds in DMSO were pin transferred into a 384-well format for screening.

### Dose-response validation

Induced motor neurons were plated in 384 well plates as they were for the screen. 20 h after plating, a 10mM test compound in DMSO was added directly to the wells using the Hewlett Packard D300 digital liquid dispenser to achieve an in-well final concentration of 10 µM, 3.3 µM, 1 µM, 333 nM, 100 nM, 33 nM, or 10 nM. Wells were backfilled with DMSO so that wells had the same amount of DMSO as the 10 µM condition, 0.1%. Cells were incubated with the test compound for 4 h at 37^o^C before the D300 was used to add 30 µM vincristine directly to the well for a final concentration of 3nM. Four replicate wells spread over two independent plates were assayed for each combination of test compound concentration and chemotherapy. After a 24-hour incubation at 37^o^C, cells were fixed and stained for β-III-tubulin. Neurite length was again quantified using the Thermo Fisher Arrayscan XTi.

### Immunocytochemistry

Motor and sensory neurons were immunostained for subtype specific markers as follows: cells were blocked in a PBS solution containing 0.3% triton and 10% donkey serum for 1.5 h at room temperature. Primary antibodies were diluted in PBS containing 0.1% triton and 3% donkey serum at 4 C overnight. The following primary antibodies were used: anti-Islet1/2 (R&D Systems, AF1837, 1:250), anti-choline acetyltransferase (Millipore, AB144P, 1:100), anti-HB9 (DSHB, 81.5C10, 1:25), anti-beta-III-tubulin (Sigma-Aldrich, T8660, 1:1000), anti-NF200 (Millipore, AB5539, 1:500), anti-peripherin (Millipore, AB1530, 1:250), and anti-Brn3a (Millipore, AB5945, 1:250). Cells were then washed 3 times for 10 min with PBS and secondary antibodies were added. The following secondary antibodies were used: donkey anti-mouse Alexa Fluor 568 (ThermoFischer, A10037, 1:500), donkey anti-goat Alexa Fluor 488 (Jackson Immuno Research, 705-545-003, 1:500), donkey anti-chicken Alexa Fluor 647 (ThermoFisher, A78952, 1:500) and donkey anti-rabbit Alexa Fluor 488 (ThermoFisher, A-21,206, 1:500) and Hoechst dye (1:10000). Secondary antibodies were incubated for 1 h at room temperature and washed 3 times for 10 min with PBS. Imaging was performed using the Molecular Devices ImageXpress Micro Confocal High-Content Imaging System.

#### Caspase assay

Caspase activation in motor neurons was measured using the Caspase-Glo 3/7 Assay System (Promega, G8090), following the manufacturer’s instructions. Caspase-Glo reagent was added in a 1:1 ratio to culture media in plate containing cells, and incubated for 30 min on a shaker at room temperature while protected from light. After incubation, luminescence was quantitated using PerkinElmer EnSight multimodal plate reader and plotted as a ratio to DMSO control.

### Statistics

Statistical analysis was performed using GraphPad Prism. For analysis of whether dose-response relations were significant, a Brown-Forsythe test was first performed to determine if groups had equal variance. If there was no difference in variance between the two groups, a one-way ANOVA was used to determine if the dose had a significant effect on a measure. A Dunnett post-hoc test was then performed to identify significant doses. Alternatively, a Kruskal-Wallis test was performed if the samples had unequal variances. *p* < 0.05 was considered significant.

## Results

### Model of vincristine-induced growth arrest in human iPSC-derived motor neurons

Historically, high-throughput screens are performed on a specific molecular target. However, these target-based screens require prior knowledge of the disease mechanism. To avoid making any assumptions regarding the mechanisms underlying CIPN, we instead set out to perform a phenotypic screen, using protection against vincristine-induced neurite length reduction as our readout. Previous work in axon regeneration and Alzheimer’s disease has validated the utility of neurite length for screens performed on iPSC-derived neurons [18] Additionally, previous studies have demonstrated a vincristine-induced neurite loss in hiPSC-derived neurons [19, 20], but phenotypic screens have not been utilized for identifying potential therapeutics for CIPN.

**Fig. 1:**
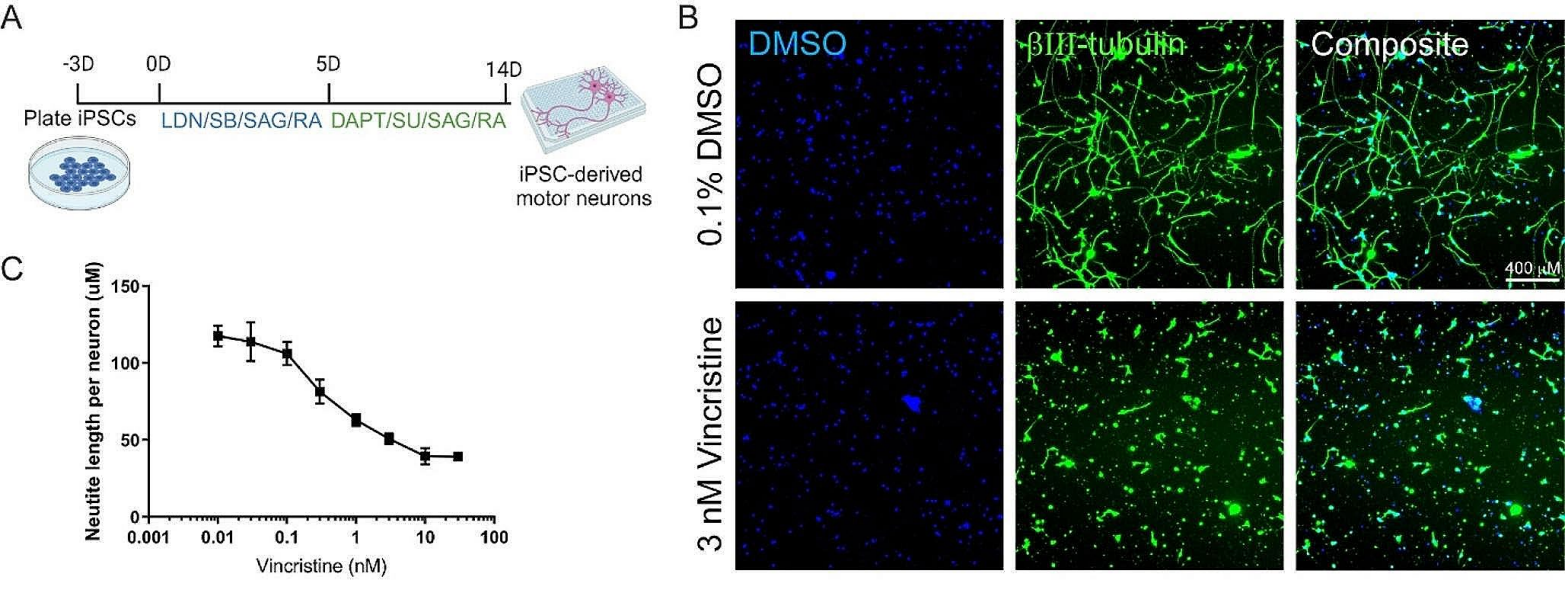
Developing a model of vincristine-induced growth arrest in human iPSC-derived motor neurons. (**A**) Timeline of human iPSC differentiation and small molecule treatment to derive induced motor neurons (iMNs). (**B**) Representative images, comparing neurites treated with 3 nM vincristine to those treated with vehicle (DMSO). Blue represents neuronal nuclei stained with DAPI, and green beta-III tubulin, an axon marker. Scale bar 400 μm. (**C**) Neurite length, measured by automated neurite tracing in iMNs treated for 24 h with escalating doses of vincristine

We have now developed an assay of vincristine-induced neurotoxicity using human iPSC-derived motor neurons, which can be used to screen for drugs that prevent vincristine’s neurotoxic effects. Human iPSCs were differentiated into Isl1, HB9, CHAT-expressing motor neurons (Suppl. Figure 1A) using a small-molecule-based differentiation method and purified based on the expression of the surface marker NCAM [15]. These human iPSC-derived motor neurons (hiMNs) were then plated on a laminin substrate for 24 h prior to exposure to varying doses of vincristine for an additional 24 h (Fig. 1A). Automated tracing software was used to measure neurite length (Fig. 1B). The hiMNs displayed a dose-dependent decrease in neurite length with increasing concentrations of vincristine (Fig. 1C).

### Small molecule screen against vincristine-induced growth arrest identifies 6 neuroprotective compounds

**Fig. 2:**
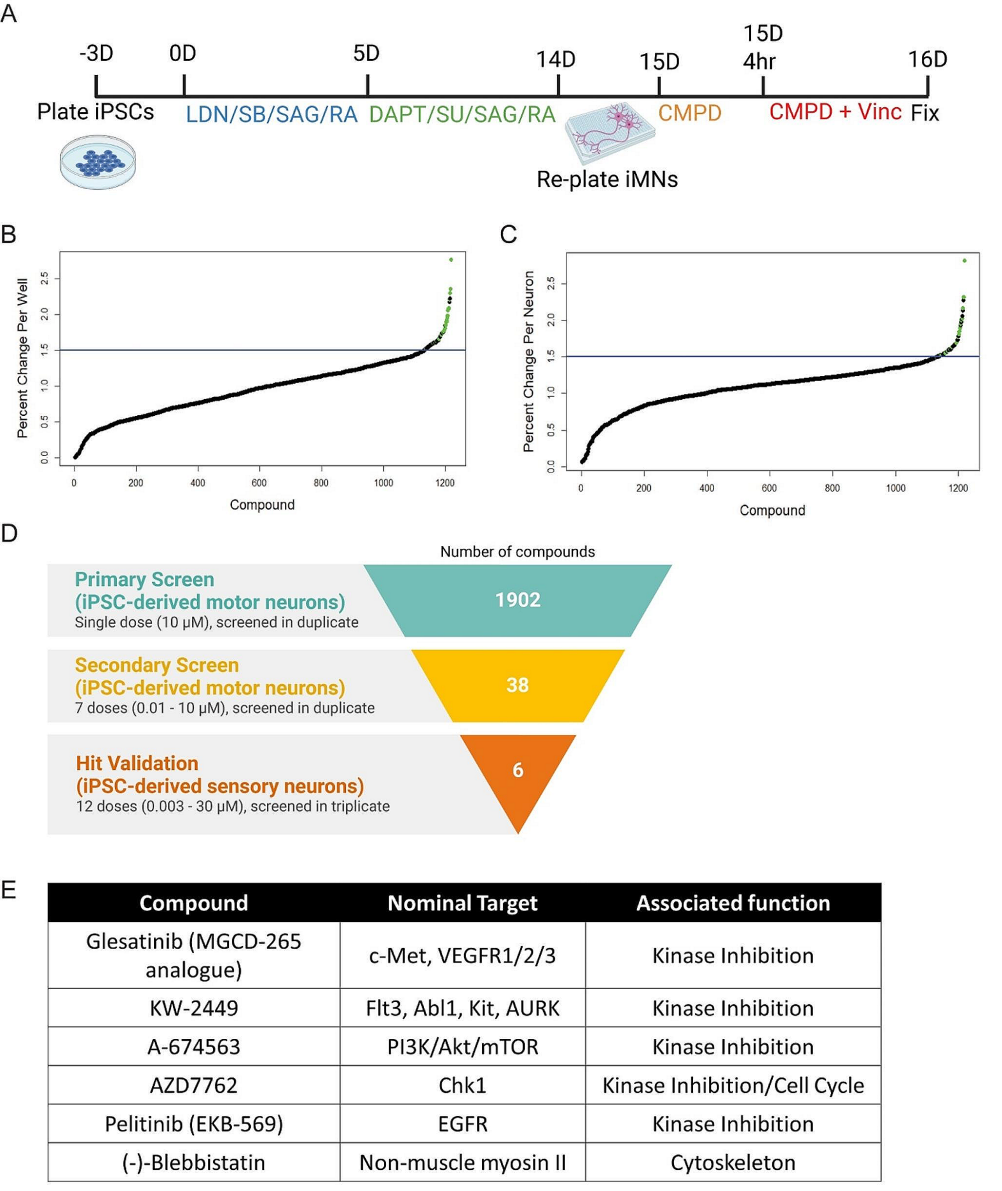
Screening for compounds that protect from vincristine-induced neurotoxicity in iMNs and iSNs. (**A**) Representation of the primary screening paradigm. Waterfall plots showing percent change in neurite length per well (**B**) and per neuron (**C**) for each of the 1902 screened compounds relative to control. Hit compounds are represented as green dots, and all other compounds as black dots. The blue line represents a 50% increase in neurite length relative to control. (**D**) Drug discovery and validation funnel diagram listing number of compounds, concentrations, neuronal cell types, and number of replicates for each stage of the screening process. (**E**) Table describing the top six compounds, their nominal targets, and associated cellular functions

To screen for drugs that prevent the neurotoxic effects of vincristine, we selected a 3 nM dose of the chemotherapeutic since it was the lowest dose which consistently resulted in a positive Z’, providing a sufficient dynamic range to perform a screen for detecting protection from vincristine-induced toxicity. This dose was reasonable when compared to plasma levels reported in patients receiving vincristine chemotherapy which range from an average of 10.7nM immediately after infusion to 0.61nM more than 12 h after an infusion [21]The screening paradigm is represented graphically in Fig. 2A. Briefly, hiMNs were plated on a laminin substrate for 20 h and then incubated in media containing 10 µM of the test compound for 4 h prior to the addition of 3 nM vincristine. The neurons were then incubated in the test-compound-vincristine mixture for 24 h prior to fixation and staining for automated quantification of neurite length.

**Fig. 3:**
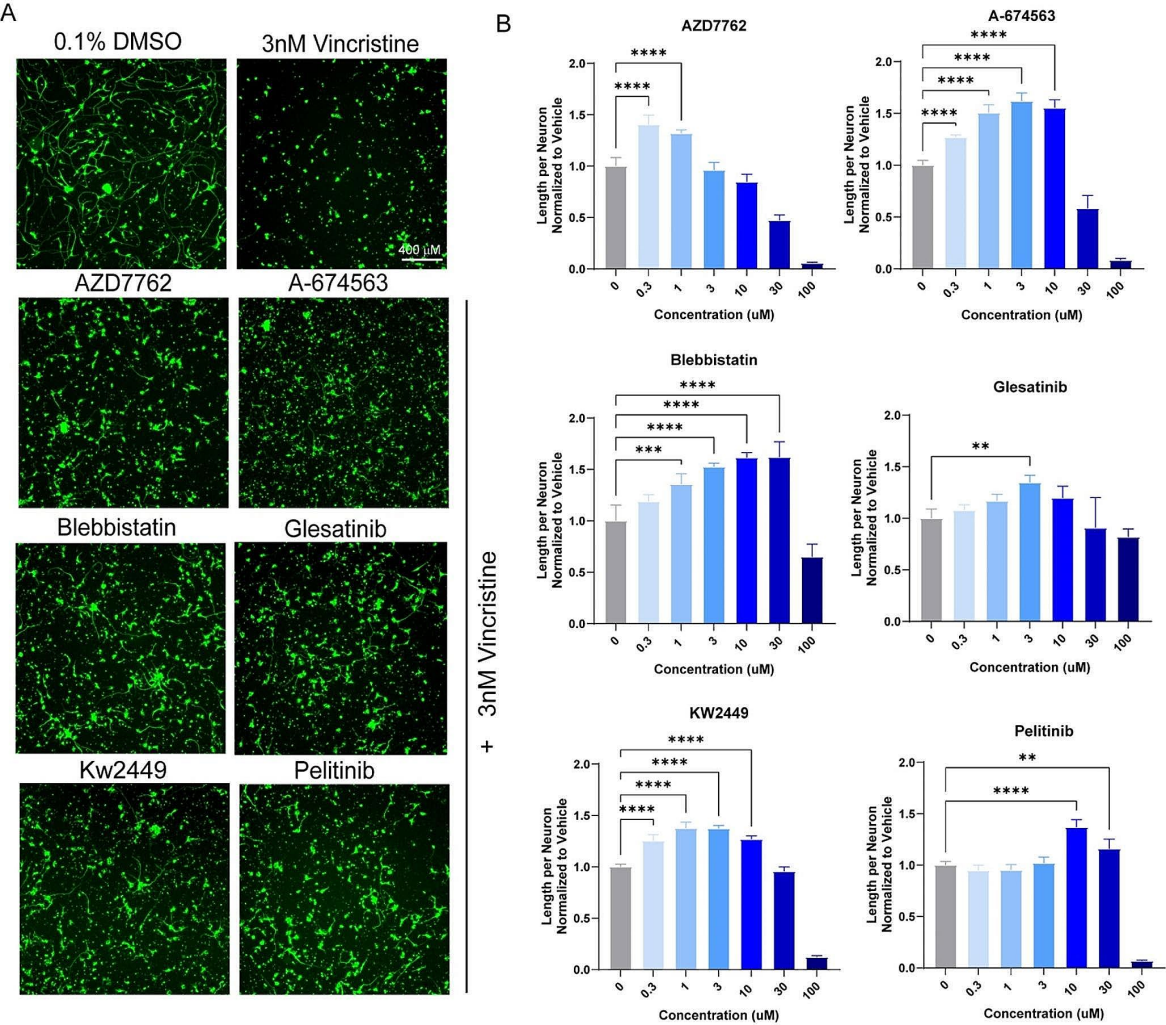
Dose-response validation of compounds identified in the primary screen. (**A**) Representative images of top six hits treated with vincristine compared to DMSO and vincristine-only wells, stained for beta-III tubulin (green). Scale bar 400 μm. (**B**) Bar graphs representing the increase in neurite length per neuron in vincristine-treated iMNs also treated with escalating doses of hit compounds. One-way ANOVA was performed for each compound. Error bars are ± SD (*n* = 5 independent experiments), **p < 0.005, ***p < 0.001, ****p < 0.0001

A total of 1902 bioactive small molecules were included in the primary screen. 38 compounds resulted in a greater than 50% increase in neurite length when quantified both on a per-well and per-neuron basis compared to control wells treated with vincristine alone (Fig. 2B and D; Table 1). All 38 compounds were then subjected to secondary screening in doses ranging from 0.3 to 100 µM (Suppl. Figure 3). Six compounds showed a promising dose-dependent rescue against vincristine-induced growth arrest – AZD7762, A-674563, Blebbistatin, Glesatinib, KW-2449, and Pelitinib (Fig. 3A and B). These compounds were then subjected to a mechanistic analysis based on published data (Fig. 2D). Five of the most neuroprotective small molecules exerted their effects through kinase inhibition, one compound affected cell cycle components, and one compound was involved in cytoskeletal function, suggesting that all of these processes might play a role in motor axon vulnerability to chemotherapy.

**Table 1:** Table listing the top 38 compounds in order of efficacy from our primary screening. Hits were selected when more than a 50% increase in neurite length per neuron was observed in each replicate. The table lists the name and the known function of the compound tested

Number	% increase neurite per neuron	Compound Name	Compound Target
1	181.09	Glesanitib (MGCD-265 analog)	VEGFR inhibitor
2	131.64	PIK-75	DNA protein kinase in hibitor|PI3K inhibitor
3	131.44	KW-2449	Abl kinase inhibitor|Aurora kinase inhibitor|FLT3 inhibitor
4	127.58	A-674563	AKT inhibitor
5	116.55	K02288	BMP inhibitor
6	102.80	AZD7762	CHK inhibitor
7	99.86	Pelitinib (EKB-569)	EGFR inhibitor
8	95.41	Alprostadil	Prostanoid receptor agonist
9	90.70	(-)-Blebbistatin	ATPase inhibitor|NMMII inhibitor
10	89.58	CP-673451	PDGFR tyrosine kinase receptor inhibitor
11	85.38	PHA-767491	CDC inhibitor
12	84.10	Batimastat (BB-94)	MMP inhibitor
13	81.12	Genistein	Tyrosine kinase inhibitor
14	80.21	Hexestrol	Synthetic estrogen
15	79.72	Sulfameter	Dihydrofolate reductase inhibitor
16	77.42	Meprednisone	Glucocorticoid
17	77.25	TAPI-1	ADAM17/TACE inhibitor
18	72.46	Naproxen	Cyclooxygenase inhibitor
19	70.53	Tenovin-1	SIRT inhibitor|TP53 activator
20	69.55	Danoprevir	HCV inhibitor
21	65.23	Ilomastat	MMP inhibitor
22	63.74	3-Deazaneplanocin A	Histone lysine methyltransferase inhibitor
23	63.67	ZCL278	CDC inhibitor
24	63.10	Lomustine	DNA synthesis inhibitor
25	60.70	CRT0044876	DNA damage
26	60.15	Dyphylline	Adenosine receptor antagonist|Phosphodiesterase inhibitor
27	59.84	Cephalexin	Bacterial cell wall synthesis inhibitor
28	59.04	Acitretin	Retinoid receptor agonist
29	58.76	SANT-1	Smoothened receptor antagonist
30	56.15	AT7519	CDK inhibitor
31	55.66	VER-49009	HSP inhibitor
32	55.59	RKI-1447	Rho associated kinase inhibitor
33	55.35	PHA-793887	CDK inhibitor
34	55.01	Sulfanilamide	Carbonic anhydrase inhibitor
35	53.34	SB-203580	TIE tyrosine kinase inhibitor
36	52.94	Rucaparib	PARP inhibitor
37	52.49	Apremilast	Phosphodiesterase inhibitor
38	50.04	Furosemide	Diuretic

In addition, we tested the top six neuroprotective compounds in a secondary assay of vincristine-induced toxicity – caspase 3/7 activation (Suppl. Figure 2). We found that 4 out of our 6 most promising compounds (AZD7762, A-674563, KW-2449 and Pelitinib) showed robust dose-dependent decrease in caspase activation. Two compounds – blebbistatin and glesatinib, did not alter caspase activation despite their robust effects on axonal outgrowth. These findings suggest that neuroprotection against vincristine-induced neurite growth arrest and caspase activation, might occur through distinct mechanisms.

### Hit compounds identified in motor neurons are neuroprotective against vincristine in iPSC-derived sensory neurons

**Fig. 4:**
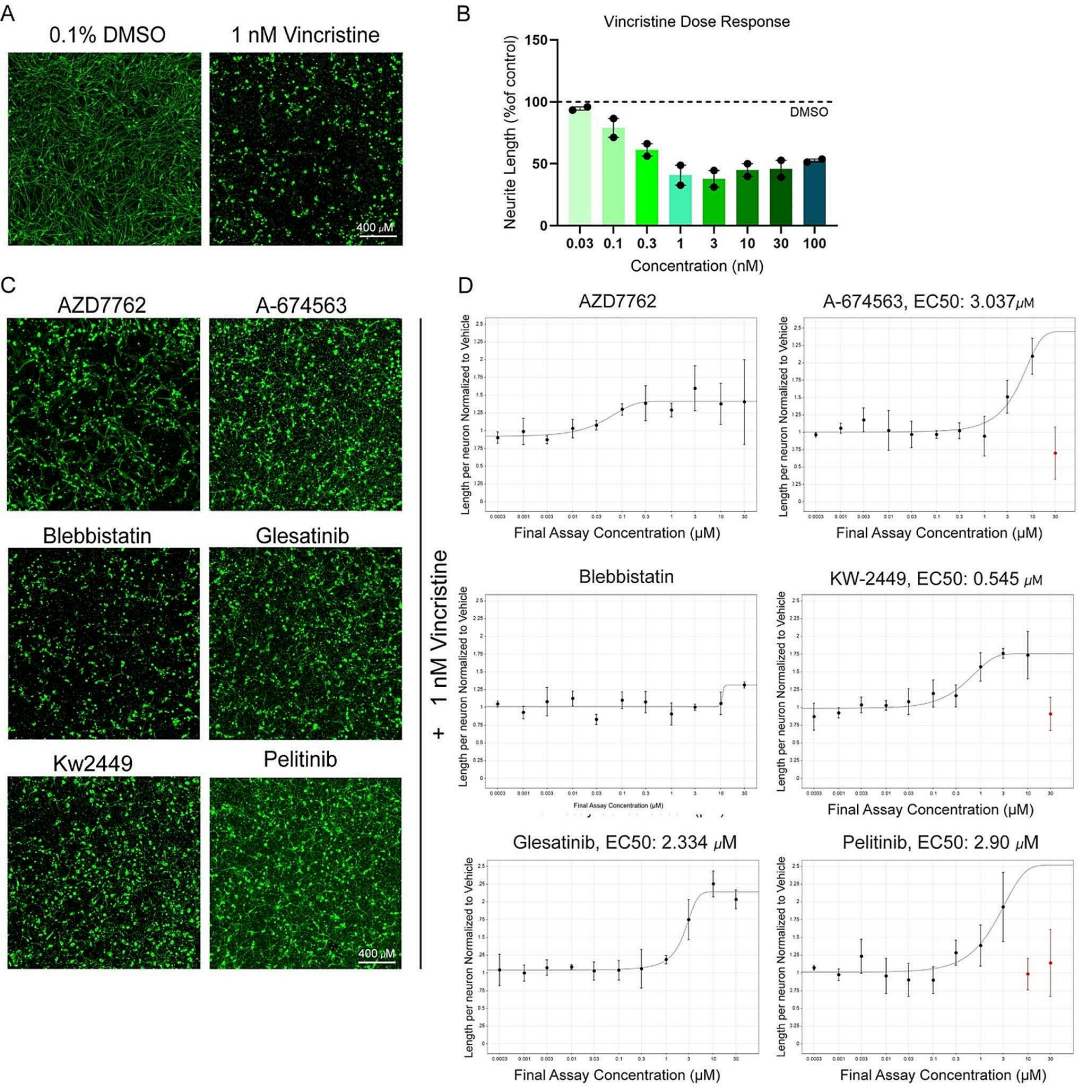
Four of the top neuroprotective compounds from the primary screen are also neuroprotective in human iPSC-derived sensory neurons. (**A**) Representative images of control (DMSO) vs. vincristine-treated sensory neurons 24 h post-treatment with cells stained for beta-III tubulin (green). Scale bar 400 μm (**B**) Bar graph showing dose-dependent reduction in neurite length upon vincristine treatment (*n* = 2 independent experiments, 4–8 wells per condition). (**C**) Representative images of sensory neurons treated with top six compounds identified from the iMN screen, in combination with vincristine, cells stained for beta-III tubulin (green). Scale bar 400 μm. (**D**) Dose response curves showing the neurite length per neuron compared to DMSO control (as %) for all six compounds. Some of the rescue compounds displayed neuronal toxicity at high doses (shown in red). Error bars represent *±* SD

Vincristine affects motor and sensory neurons in the peripheral nervous system. To validate whether the compounds identified in the motor neuron screen are also neuroprotective in sensory neurons, we utilized a protocol to generate human iPSC-derived sensory neurons which express the sensory neuron markers – peripherin and Brn3a (Suppl. Figure 1B) [16]. These neurons were treated with increasing doses of vincristine to find the lowest dose of vincristine which causes significant decrease in neurite outgrowth. Vincristine was toxic in a dose-dependent manner with 1 nM vincristine causing reliable decrease in neurite outgrowth (Fig. 4A and B). Sensory neurons were then plated on a laminin substrate for 20 h before the rescue compounds were administered for 4 h, and then 1 nM of vincristine was applied for a further 24 h before fixation. Neurons were then stained for the neuronal marker beta-III tubulin, and neurite length was measured using automated software. We performed an 11-point dose response to test the rescue properties of our top six compounds identified from motor neuron screens in order to account for any differential efficacy between the two neuronal cell types. Four of the six compounds (A-674563, Glesatinib, KW-2449, and Pelitinib) were neuroprotective against vincristine-induced neurite growth arrest in this model, exhibiting EC50s between 0.5 and 3 µM (Fig. 4C and D). Two of the compounds (Blebbistatin and AZD7762) did not show a dose-dependent rescue against vincristine and we were unable to reliably calculate EC50s for these compounds. These results indicate that our approach enabled us to identify small molecules that are neuroprotective in both sensory and motor neurons, however there are also neuron type-specific neuroprotectants, which could be identified with extensive future screening efforts.

## Discussion

We have developed a novel, high-throughput screening platform using human-derived motor neurons for the identification of neuroprotective, bioactive small molecules against vincristine-induced neurotoxicity. The platform involves the use of 384-well plates, automated liquid handling devices, and an automated neurite tracking algorithm, which allows for the screening of hundreds of compounds simultaneously in a systematic and unbiased manner. This system allowed us to screen 1902 bioactive compounds in duplicate and enabled us to identify 38 small molecules, that increased neurite length by 50% compared to vincristine treatment alone, a hit rate of 2%. In secondary screens, we tested each of the 38 compounds in a 7-point dose-response experiment and identified six compounds that consistently showed a dose-dependent rescue from vincristine-induced neurite growth arrest. Four out of our top six neuroprotective compounds in motor neurons were also neuroprotective in a dose-dependent manner in iPSC-derived sensory neurons.

Interestingly, five compounds from the most promising hits alter kinase function (Fig. 2C). Kinases are a specialized set of enzymes primarily involved in the phosphorylation of cellular substrates. They also act like scaffolds of protein complexes, alter DNA binding, and have allosteric effects on other enzymes and subcellular targets [22]. Kinase inhibition protects motor neurons from chemically induced neurotoxicity in models of amyotrophic lateral sclerosis [23–25]. Future studies identifying which specific kinases play a role in vincristine-induced growth arrest in motor neurons will be of interest. Other molecular mechanisms that emerged from the hits of this screen include cell cycle-related processes and cytoskeletal function, which have previously been implicated in neurodegeneration and chemotherapy-induced toxicity [26–29].

Vincristine-induced peripheral neuropathy is characterized by both motor and sensory symptoms, indicating that the systemic administration of chemotherapy affects both sensory and motor axons in the periphery despite their distinct cell body location, morphology, and function. For example, motor neuron cell bodies reside within the ventral horn of the spinal cord, are multipolar with a single axon and multiple dendrites and innervate muscles. On the other hand, sensory neuron cell bodies reside within the dorsal root ganglion, have a pseudounipolar morphology, and transmit sensory information from the periphery to the central nervous system. Interestingly, four out of the six most neuroprotective compounds in our motor neuron model – KW-2449, A-674563, Glesatinib and Pelitinib - also showed neuroprotection in a sensory neuron model of vincristine-induced growth arrest, which points to common mechanisms of neuroprotection across distinct subtypes of peripheral neurons. The compounds showed similar neuroprotection efficacy in motor and sensory neurons with EC50s in the low micromolar ranges and the extent of rescue was 50 − 100% increase in neurite length compared to neurons treated with vincristine alone. Two compounds – blebbistatin and AZD7762, did not show a reliable dose-dependent rescue in sensory neurons, suggesting that there are also neuron subtype specific processes taking place, and more extensive screening needs to be utilized in the future to capture these differences.

Despite the fact that the prevalence of sensory symptoms is higher than motor symptoms in CIPN patients, we chose to use motor neurons for our primary and secondary screens for several reasons. We had access to a robust motor neuron differentiation protocol, that was previously validated and described in peer-reviewed literature [15, 30] This protocol produces a large number of pure motor neurons, that are suitable for high-throughput screening. There is a possibility that some sensory neuron-specific neuroprotectants were not identified due to our screening strategy. Future primary and secondary screens in sensory neurons will be required to expand the selection of neuroprotective compounds to include those specific to sensory neurons.

In addition, we used a secondary measure for vincristine-induced toxicity in motor neurons – caspase activation. We found that four out of six of our most protective compounds in the neurite outgrowth paradigm, also prevented caspase activation (Suppl. Figure 2). Interestingly, two compounds – blebbistatin and glesatinib, had no effect on the caspase activation. These findings suggest that vincristine-induced growth arrest and caspase upregulation might occur through distinct molecular mechanisms, where some compounds are able to attenuate both, and others have differential effects. In future studies, the screening paradigm could be expanded to perform neurite outgrowth and caspase activation primary rescue screens to contrast and compare the individual compound activity in both assays. Another consideration for future experiments should be the age, sex and race of the iPSC line donors. In this study, we used two different iPSC lines – one derived from a 46-year-old female and one from < 1 day old male. Although we cannot absolutely rule out that some of the differential compound activity across neuronal subtypes is due to genetic and environmental factors across these lines, we were still able to robustly identify small molecules that are neuroprotective against vincristine in neurons derived from both lines.

Some of the compounds identified as our top six neuroprotective hits from the iPSC-motor neuron screen against vincristine-induced growth arrest have previously been identified as neuroprotective or pro-regenerative. For example, KW-2449 ameliorates Rett’s Syndrome disease pathology in mice [31] as well as human-derived neuronal and organoid models of that disease [32]. Despite their use in anti-cancer therapy, glesatinib, and pelitinib regulate the expression of neuroprotective miRNA as detected in a large screen of human stem cell-induced neurons [33]. Blebbistatin is a non-muscle myosin inhibitor that promotes neuronal outgrowth on inhibitory extracellular substrates [34, 35]. It has also been recently shown to promote axon regeneration after laser axotomy and was identified as a top neuroregenerative compound in a high-throughput screen in human iPSC-derived motor neurons [36]. Interestingly, Blebbistatin did not protect sensory neurons against vincristine-induced growth arrest in our model. AZD7762 was highly neuroprotective against vincristine in our iPSC-derived motor neurons model but only showed mild neuroprotection in sensory neurons. AZD7762 is a Chk1/2 inhibitor that is utilized as an enhancer of chemotherapy-induced apoptosis of cancer cells; however, its effects on neurons have not been widely studied [37] and the target for this compound in neurons needs to be identified.

Any compound identified as neuroprotective needs to be assessed for its effect on chemotherapy-induced toxicity in cancer cells to ensure that neuroprotectants do not interfere at all with the killing of cancer cells and tested for any deleterious side effects. Several biomarkers have recently been identified as predictors of a higher risk of developing CIPN in cancer patients undergoing chemotherapy, including neurofilament light chain [38, 39]. Ideally, if such markers are universally implemented for patients undergoing chemotherapy, those patients identified as being at high risk for developing CIPN could benefit from treatment with a neuroprotective compound prior to the onset of their chemotherapy regime. Effective treatment with neuroprotective compounds will also depend on their ability to rescue neuronal toxicity and axonal growth defects produced by other chemotherapy agents, such as taxanes or platinum agents, which would also advance our knowledge of the disease mechanisms driving CIPN.

In conclusion, human-derived motor neurons can be used in a high-throughput platform to screen for neuroprotective small molecules against the growth arrest defects induced by chemotherapy treatment. Our screen of 1902 bioactive molecules identified six promising candidates - AZD7762, A-674563, Blebbistatin, Glesatinib, KW-2449, and Pelitinib, that all produce a dose-dependent rescue of vincristine-induced toxicity in motor neurons and four of those were protective of sensory neurons too. These results show that this approach could aid in the identification of novel neuroprotectants against chemotherapy-induced neuronal toxicity for both sensory and motor neurons, which could be evaluated as therapeutics to protect against the development of CIPN.

### Electronic Supplementary Material

Below is the link to the electronic supplementary material.

Supplementary Material 1: Supplementary Fig. 1. Motor and sensory neuron identity validation. (A) Representative immunocytochemistry images of iMNs at 15 DIV, stained for motor neuron-specific markers (Islet1/2, HB9 and CHAT) and neuronal markers (NF200, beta-III-tubulin). Scale bar is 50 μm. (B) Representative immunocytochemistry images of iSNs at 15DIV, stained for sensory neuron-specific markers (Peripherin, Brn3a) and neuronal markers (NF200, beta-III-tubulin). Scale bar is 100 μm; Supplementary Fig. 2. Four out of our six most promising compounds also attenuate vincristine induced caspase activation (A) Dose response curves showing the average ratio of caspase 3/7 activation compared to DMSO control for our top six most promising neuroprotective compounds in motor neurons. Error bars represent *±* SD; Supplementary Fig. 3. Dose response for top 38 neuroprotective compounds identified in the primary screen. (A) Top six compounds which showed most promising dose-dependent neurite outgrowth rescue in motor neurons in primary and secondary screens. (B) Dose response curves for the rest of the 38 top compounds identified in primary screens, upon secondary screening in motor neurons in an 8-point dose response.

## Data Availability

The data that support the findings of this study are available from the corresponding author upon reasonable request.

## Abbreviations

CIPN: chemotherapy-induced peripheral neuropathy
iPSC: induced pluripotent stem cells
JNK: c-Jun N-terminal kinase
hiMNs: human induced motor neurons
NCAM: neural cell adhesion molecule
