# Supplementary material for: Identification of novel neuroprotectants against vincristine-induced neurotoxicity in iPSC-derived neurons
Source: Cell Mol Life Sci. 2024 Jul 27;81(1):315. doi: 10.1007/s00018-024-05340-x (PMC11335239; doi:10.1007/s00018-024-05340-x)
Supplement: Supplementary file 1 — Supplementary Material 1: Supplementary Fig. 1. Motor and sensory neuron identity validation. (A) Representative immunocytochemistry images of iMNs at 15 DIV, stained for motor neuron-specific markers (Islet1/2, HB9 and CHAT) and neuronal markers (NF200, beta-III-tubulin). Scale bar is 50 μm. (B) Representative immunocytochemistry images of iSNs at 15DIV, stained for sensory neuron-specific markers (Peripherin, Brn3a) and neuronal markers (NF200, beta-III-tubulin). Scale bar is 100 μm; Supplementary Fig. 2. Four out of our six most promising compounds also attenuate vincristine induced caspase activation (A) Dose response curves showing the average ratio of caspase 3/7 activation compared to DMSO control for our top six most promising neuroprotective compounds in motor neurons. Error bars represent ± SD; Supplementary Fig. 3. Dose response for top 38 neuroprotective compounds identified in the primary screen. (A) Top six compounds which showed most promising dose-dependent neurite outgrowth rescue in motor neurons in primary and secondary screens. (B) Dose response curves for the rest of the 38 top compounds identified in primary screens, upon secondary screening in motor neurons in an 8-point dose response. [file 18_2024_5340_MOESM1_ESM.docx]

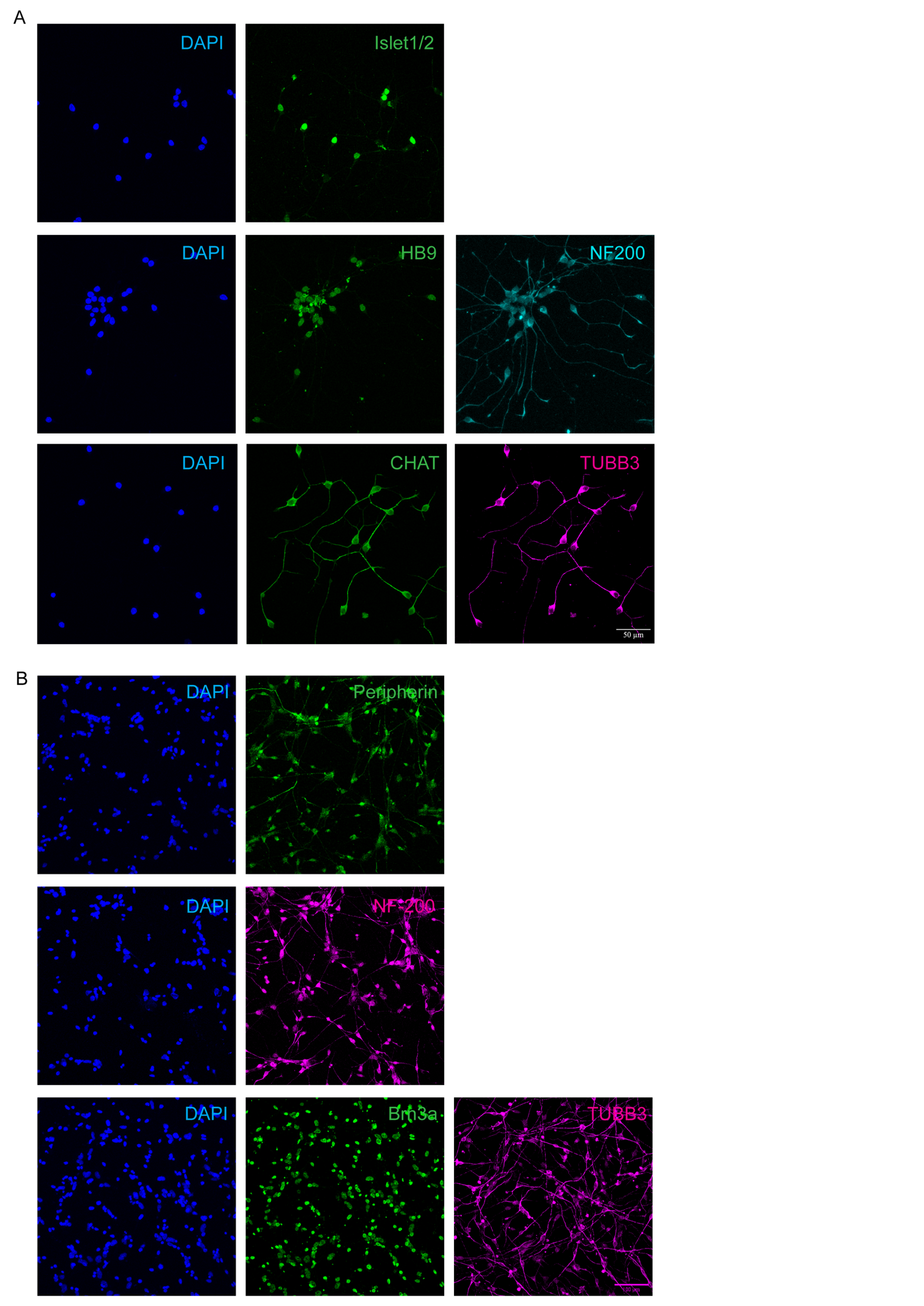


**Supplementary Figure 1** Motor and sensory neuron identity validation. (A) Representative immunocytochemistry images of iMNs at 15 DIV, stained for motor neuron-specific markers (Islet1/2, HB9 and CHAT) and neuronal markers (NF200, beta-III-tubulin). Scale bar is 50 μm. (B) Representative immunocytochemistry images of iSNs at 15DIV, stained for sensory neuron-specific markers (Peripherin, Brn3a) and neuronal markers (NF200, beta-III-tubulin). Scale bar is 100 μm.


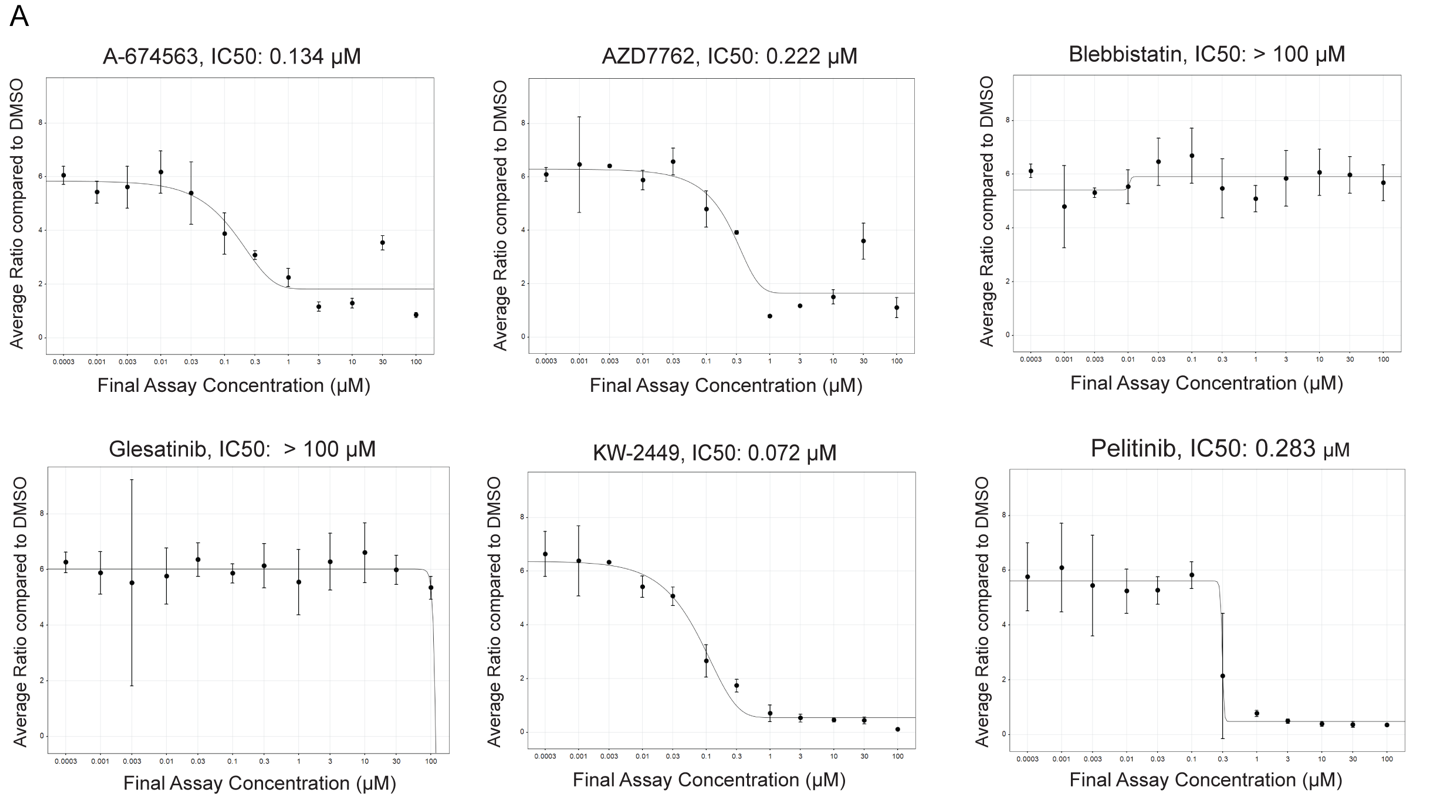


**Supplementary Figure 2** Four out of our six most promising compounds also attenuate vincristine induced caspase activation (A) Dose response curves showing the average ratio of caspase 3/7 activation compared to DMSO control for our top six most promising neuroprotective compounds in motor neurons. Error bars represent + SD.

**
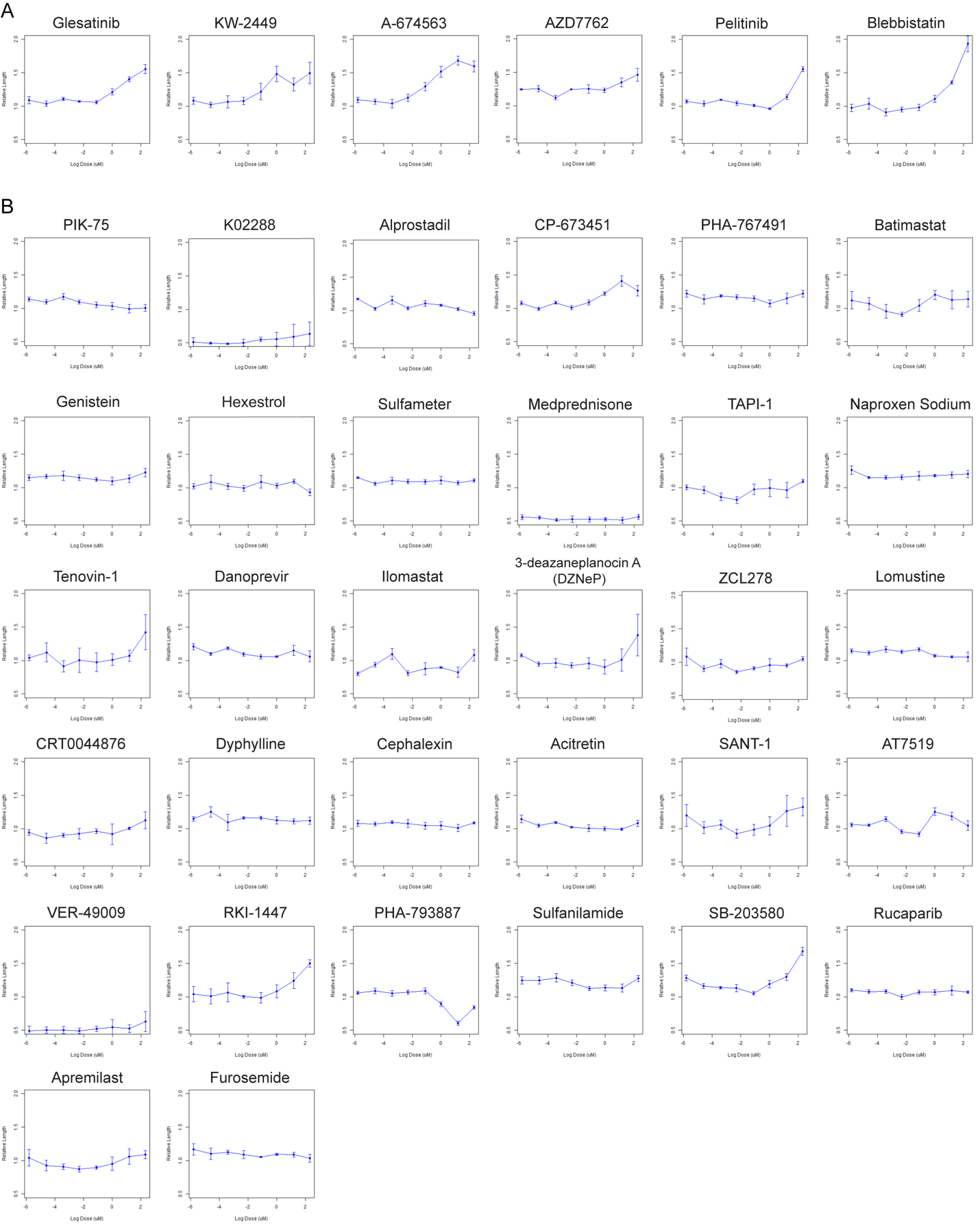
**

**Supplementary Figure 3** Dose response for top 38 neuroprotective compounds identified in the primary screen. (A) Top six compounds which showed most promising dose-dependent neurite outgrowth rescue in motor neurons in primary and secondary screens. (B) Dose response curves for the rest of the 38 top compounds identified in primary screens, upon secondary screening in motor neurons in an 8-point dose response.
